# “Nano-ghosts”: Risk assessment of submicron-sized particles in food biased towards fictional “nano”

**DOI:** 10.17179/excli2022-4630

**Published:** 2022-01-13

**Authors:** Hanspeter Naegeli, Corina Gsell

**Affiliations:** 1 Institute of Pharmacology and Toxicology, University of Zurich-Vetsuisse, Zurich, Switzerland

**Keywords:** food additive, titanium dioxide, micron-sized particles, aggregate, agglomerate

## Abstract

Much confusion has been generated in the safety assessment of food-grade TiO_2_ (E171) by the comingling of studies conducted on submicron-sized particles with those examining the toxicity of more minuscule counterparts. As E171 displays a nano-sized tail in its particle distribution (up to 36 % of particles with a diameter < 100 nm), it was thought that potential hazards of this food additive can be extrapolated from studies on thoroughly nanoscale formulations. This simplistic procedure may, however, overestimate the effects of the nano-sized tail of E171 because TiO_2_ particles readily aggregate or agglomerate in aqueous suspensions and biological matrices. The resulting larger clusters display a reduced oral bioavailability in comparison to the same material in nano-sized dimensions. Also, even if taken up in trace amounts, the smaller particles likely remain appended to larger particles or clusters and these aggregates or conglomerates may nullify to a great extent their “nano” characteristics. The purpose of this review is, therefore, to reevaluate the literature on the toxicity of TiO_2_ particles focusing on studies that are directly relevant for the assessment of E171. The purpose is not to avert a ban on the use of E171 in food, which might well be justified in light of the uncertainties associated with this additive employed solely for its colorant properties. Instead, it will be important to avoid in the future this same bias towards a fictional “nano” hazard, especially when evaluating more innovative engineered particles that confer true benefits for example by enhancing nutritional properties, quality, freshness, traceability or sustainability of food.

## 1. Introduction

Submicron-sized TiO_2_ is used since 1969 as a white pigment in many food products including candies, pastries, coffee cream powders, salad dressings, sauces and sandwich spreads (Weir et al., 2012[82]; Peters et al., 2014[58]; Rompelberg et al., 2016[65]). In the US, the Food and Drug Administration permits up to 1 % by weight of TiO_2_ as a food colorant (FDA, 2021[26]). In the EU, TiO_2_ was until recently an authorized food additive, listed as E171, to be used at *quantum satis*, i.e., with no upper limit as long as the levels are not higher than what is needed for the intended scope (Regulation (EC) No. 1333, 2008[62]). According to use levels reported by food industry, highest concentrations of TiO_2_ are to be expected in chewing gums (up to 16000 mg/kg) as well as in foods (such as for example processed nuts) and food supplements marketed in a solid form (ANS Panel, 2016[3]). The dietary consumption of food-grade TiO_2_ in Western populations has been calculated at around 1-10 mg/kg body weight per day with higher exposure in children compared to adults (Weir et al., 2012[82]; Bachler et al., 2015[5]; ANS Panel, 2016[3]; Rompelberg et al., 2016[65]; Sprong et al., 2016[70]).

In 2021, the Panel on Food Additives and Flavorings (FAF Panel) at the European Food Safety Authority (EFSA) published a reassessment of E171 as food additive (FAF Panel, 2021[24]). In this latest scientific opinion, the experts of the FAF Panel raised doubts as to the safety of E171 and concluded that, based on newer studies, a possible genotoxicity of E171 could no longer be ruled out. This genotoxicity has been related to a subfraction of E171 with primary particle dimensions in the nanomaterial scale (see section 2). The European Commission and Members States subsequently proposed withdrawing E171 from the list of additives permitted within the EU. Following the example of France, where E171 is already prohibited since the beginning of 2020, a European ban on the use of E171 as food additive is likely to come into effect and to be enforced after a certain transition period. The purpose of the present review is not to contradict the conclusions of the FAF Panel as to existence of uncertainties associated with the safe use of E171. However, it is important to discuss the gaps and weaknesses of the current risk assessment approach to avoid that, in the future, innovative and more beneficial particulate food additives (serving for example to enhance nutritional properties, for the detection of pathogens or as markers for freshness and traceability) may fail market approval in the EU just because of an alleged “nano” connotation.

## 2. Physico-Chemical Properties of E171

Titanium (Ti) is an abundant element in the earth's crust and TiO_2_ constitutes its naturally occurring oxide (Diebold, 2003[21]; Theissmann et al., 2014[74]). TiO_2_ minerals contain impurities that confer various intrinsic colors. Instead, manufactured food- and pigment-grade formulations appear as a white powder that, in pure form, assemble in three different types of crystals, i.e., anatase, rutile (with tetragonal coordination of Ti atoms) and brookite (with rombohedral coordination of Ti atoms). Only anatase and rutile (or mixtures of these two structures) are employed in food (Regulation (EU) No. 231, 2012[63]). The surface of anatase particles is considered more reactive than that of rutile, as evidenced by the higher ability to generate reactive oxygen species (ROS) in aqueous solutions (Sayes et al., 2006[67]) and to stimulate cells of the innate immune system (Vandebriel et al., 2018[77]). However, surface properties may be modulated by coating with a layer of alumina or silica (Regulation (EU) No. 231, 2012; JECFA, 2012[63]).

The size of anatase/rutile particles is a critical parameter to achieve the desired colorant effect. In fact, E171 ideally displays a diameter of approximately half the wavelength of the light to be scattered, i.e., half of the 400-700 nm wavelength of the visible light spectrum (Mie, 1908[49]). Thus, the scattering of light by TiO_2_ and its property as a white colorant is maximized with diameters of 200-300 nm. Smaller particles are not suited because they become transparent when their size approaches the “nano” range. A European Commission Recommendation defined nanomaterials as natural, incidental or manufactured materials containing 50 % or more of the particles, determined in a number-based size distribution, with at least one external dimension not exceeding 100 nm (Commission EU, 2011[18]). Inherent to the E171 production process, there is always a broad size distribution comprising particles with a primary size below 100 nm even when the mean diameter is in the submicron target range. Distribution analyses of different food-grade TiO_2_ samples by transmission electron microscopy (TEM) showed that nano-sized particles usually occur at a frequency between 17 % and 36 % by number (Weir et al., 2012[82]; Yang et al., 2014[84]).

**Figure 1:**
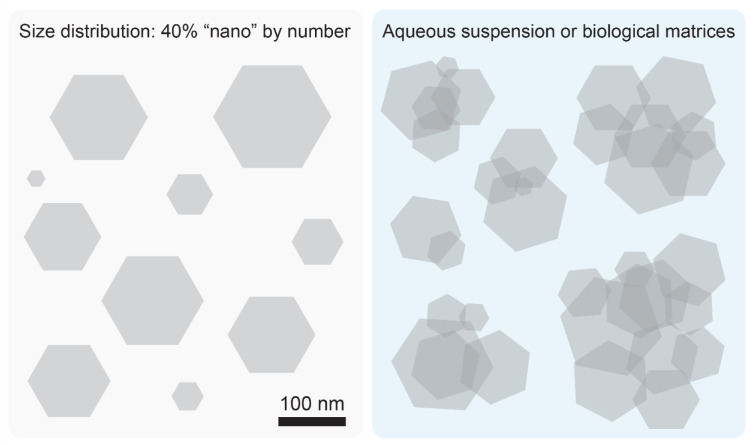
E171 assembles into particle clusters. Scheme illustrating how a population of particles consisting of 40 % “nano” by number (panel on the left; mean diameter of particles: 125 nm) forms larger clumps by aggregation/agglomeration in an aqueous suspension or biological matrices (panel on the right), thus nullifying the “nano” properties of the material.

Clearly, a reduction in size changes the overall material characteristics. Nano-sized particles display an increased surface-to-mass ratio that enhances their reactivity (Oberdörster, 2000[53]; Donaldson et al., 2001[22]) and, in addition, they easily penetrate biological barriers including the gastrointestinal mucosa (Brun et al., 2014[10]; Gitrowski et al., 2014[28]). However, the presence of a “nano” fraction in E171 does not make this material automatically harmful, because both anatase and rutile particles tend to aggregate and/or agglomerate to form larger particle clumps. Disruption of these complexes by forceful sonication is usually needed to visualize the nano-sized particle tail (Yang et al., 2014[84]; Peters et al., 2014[58]; Murugadoss et al., 2020[50]; Bischoff et al., 2021[7]). The term “aggregate” denotes an assembly of particles held together by covalent or metallic bonds, whereas “agglomerates” result from weak forces such as van der Waals interactions, hydrogen bonds, electrostatic attractions or adhesion by surface tension. Thus, even though E171 formulations contain a nanoparticle tail in their size distribution, when present in an aqueous milieu or biological matrix like food, gastrointestinal lumen, blood or internal tissues, these smaller TiO_2_ particles in the “nano” range aggregate/agglomerate with themselves and, importantly, with the concomitantly present larger particles, such that the resulting clusters lose nanomaterial properties (Yang et al., 2014[84]; Donner et al., 2016[23]; Urrutia-Ortega et al., 2016[76]; Coméra et al., 2020[17]). If a nano-sized particle associates with a submicron-sized counterpart, the “nano” characteristic of that particle vanishes (Figure 1(Fig. 1)). From these considerations it can be concluded that the “nano” component of E171 is possibly a fictional entity whose biological effect, if any, is mostly buffered by adsorption to submicron-sized particles or particle clusters. Consequently, any hazard extrapolation from nano-sized TiO_2_ materials, lacking such a submicron-sized component, may be irrelevant for the safety assessment of E171.

## 3. Peculiarity of Particulate Food Additives

Another level of complexity is the presence of surface-bound molecules altering outside properties (Nel et al., 2009[52]; Ruh et al., 2012[66]; Walkey and Chan, 2012[78]). In fact, TiO_2_ particles avidly adsorb on their surface surrounding molecules including proteins that modify fundamental characteristics including particle diameter, aggregation or agglomeration state, interactions with cell membranes, bioavailability, systemic distribution and adverse effects. The term of “corona” was introduced to denote the attachment of macromolecules from a given biological matrix to the surface of nanoparticles (Cedervall et al., 2007[12]; Lundqvist et al., 2008[43]). The particles of E171 are readily decorated with food ingredients, components of the gastrointestinal juice and, after intestinal absorption, plasma proteins (McClements et al., 2016[47]; DeLoid et al., 2017[20]). The extent of such molecular interactions depends on the surface chemistry, coating and diameter of the particles. A highly curved outer surface increases the deflection angle between absorbed macromolecules, possibly leading to a higher density of macromolecules in the corona of minuscule particles compared to larger ones. These considerations highlight, on the one hand, the importance of a thorough particle characterization for an appraisal of their effects in biological systems. This review, therefore, only includes studies in which test materials were characterized at least with respect to shape, crystalline form, particle size (preferably with measurements of the primary particle diameter), surface coating and aggregation/agglomeration state. Table 1(Tab. 1) (References in Table 1: Chakrabarti et al., 2019[13]; Hashem et al., 2020[32]; Medina-Reyes et al., 2020[48]; Orazizadeh et al., 2014[55]; Sycheva et al., 2011[71]; Urrutia-Ortega et al., 2016[76]; Wang et al., 2007[79]) provides examples of studies that were excluded due to missing information on the physico-chemical properties of test materials. On the other hand, the usual delivery, by intragastric gavage of aqueous suspensions, may not reproduce the biological effects of particles and their corona once incorporated into food (Blevins et al., 2019[8]). While gavage is a route of choice for the testing of medicinal products, it should be taken into account in future studies that this mode of administration might be less fit-to-purpose to test the safety of particulate food additives.

## 4. Uptake after Oral Ingestion

This review starts by summarizing toxicokinetic studies, including those carried out with TiO_2_ nanoparticles, to highlight differences in the bioavailability depending on the material size. It was shown that > 99 % of orally ingested TiO_2_ particles transit the gastrointestinal tract and are passed to the feces without any substantial dissolution or absorption. Only a small fraction of the particles is retained in intestinal crypts (Powell et al., 2010[61]; Brun et al., 2014[10]; Coméra et al., 2020[17]). Reports in the literature converge on the finding that some of these indigestible particles, once retained in the intestinal mucosa, can move into the intestinal wall underlying the epithelial layer and, although in very small amounts, to the blood, the liver and other organs (Ammendolia et al., 2017[2]; Bettini at al., 2017[6]; Talamini et al., 2019[72]). The translocation through epithelial cells of the intestinal tract is not efficient, but another route of uptake is through the phagocytic microfold cells (M-cells) of the mucosa, whose function is to deliver food-borne particles to the lymphoid tissue of Peyer's patches (Jani et al., 1994[38]; Hussain et al., 2001[36]; Riedle et al., 2020[64]). In this gut-associated lymphoid tissue, particles accumulate as pigment depositions (Powell et al., 1996[60], 2010[61]; Hummel et al., 2014[35]) and, through lymphatic vessels, these particles may ultimately reach the blood. Translocation of nanoparticles from the intestinal lumen to the submucosal tissue also occurs through mucus-secreting Goblet cells (Brun et al., 2014[10]). Moreover, dendritic cells can internalize nano- and micro-sized particles (Manolova et al., 2008[46]).

Oral exposure studies have been conducted in rodents both by gavage and, in few cases, by alternative oral deliveries. Table 2(Tab. 2) (References in Table 2: Ammendolia et al., 2017[2]; Bettini et al., 2017[6]; Blevins et al., 2019[8]; Cho et al., 2013[16]; Coméra et al., 2020[17]; FAF Panel, 2021[24]; Farrell and Magnuson, 2017[25]; Geraets et al., 2014[27]; Gu et al., 2015[30]; Hendrickson et al., 2016[33]; Jani et al., 1994[38]; Kreyling et al., 2017[41]; MacNicoll et al., 2015[33]; National Toxicology Program, 1979[51]; Riedle et al., 2020[64]; Talamini et al., 2019[72]; Tassinari et al., 2014[73]; Warheit et al., 2015[81][80]) summarizes the toxicokinetic and toxicodynamic studies discussed in this review. A read-across from inhalation or dermal exposure is not useful because the lining of the gastrointestinal tract is structurally and functionally different from the permeable and reactive alveolar epithelium of the lung and the tight *stratum corneum* barrier of the skin.

### 4.1 Oral bioavailability and biodistribution in rats

A quantitative toxicokinetic study was conducted by taking advantage of a vanadium (V) radiotracer method. Briefly, anatase was irradiated with a proton beam to generate radiolabeled [^48^V]TiO_2_ that forms spherical aggregates/agglomerates with a mean diameter of ~60 nm. It was then demonstrated that most ^48^V ions remain associated with the TiO_2_ particles under conditions mimicking the milieu of the gastrointestinal tract. An aqueous suspension of this test material (30-80 µg/kg body weight) was administered by gavage to Wistar-Kyoto rats (Kreyling et al., 2017[41]). Groups of 4 animals were sacrificed 1, 4, 24 h and 7 days after this single dose to determine the transfer of [^48^V]TiO_2_ from the gastrointestinal tract into blood, urine, feces and organs. Most radioactivity was excreted with the feces but, at 1 h after gavage, a small proportion of radioactivity (~ 0.6 %) was detected in the blood and internal organs (liver, spleen and kidneys). The proportion of radioactivity retained in interior compartments gradually dropped to ~ 0.05 % after 7 days.

In an earlier single dose study, Sprague-Dawley rats (6 animals per treatment group) fed with Ti-free diet received by gavage TiO_2_ (5 mg/kg body weight) as an aqueous suspension of nano- or micron-sized particles with mean diameters ranging from 40 nm to 5 µm (MacNicoll et al., 2015[44]). These particles consisted of anatase or rutile. Ti levels were measured by inductively coupled plasma (ICP)-mass spectrometry (MS) in the feces, blood and urine at different times after administration, but no Ti translocation from the gastrointestinal tract into blood or urine was observed. The animals were sacrificed 4 days after gavage for tissue analysis, which demonstrated that Ti concentrations in liver, spleen, kidney and other internal organs remained in the control range of untreated animals.

In a short-term study, an aqueous suspension of anatase nanoparticles (with a size range of 20-60 nm) was administered by gavage to Sprague-Dawley rats (14 animals per group) at doses of up to 2 mg/kg per day for five consecutive days (Tassinari et al., 2014[73]). Thereafter, ICP-MS revealed a slight increase of Ti concentrations in the spleen and, surprisingly, in the ovaries of animals exposed to the highest dose relative to untreated controls. These measurements reflect Ti levels and, hence, it is not clear whether the Ti detected in the body is due to translocation of TiO_2_ in the form of particles or as solubilized material. The authors, therefore, combined single-particle ICP-MS with scanning electron microscopy (SEM) for the analysis of tissue homogenates to demonstrate penetration of particles into the spleen (but not into the ovaries). Also, this analysis revealed that the internalized particles cluster into aggregates/agglomerates with diameters in the sub-micrometer range. In its recent opinion, the FAF Panel at EFSA concluded that approximately 0.001 % by weight of the administered particles appeared in the spleen and ovaries (FAF Panel, 2021[24]). The same experimental design was repeated by Ammendolia et al. (2017[2]) using the identical test material, with the exception that the treatment groups were increased to 20 animals. After 5 days of administration, sections of the small intestinal tissue obtained from 4 animals per group were thoroughly washed with water and analyzed for their Ti content by ICP-MS. These analyses revealed a slight but statistically significant increase of Ti concentrations relative to untreated controls in the small intestinal wall of animals exposed to the highest dose of 2 mg/kg. The FAF Panel (2021[24]) calculated that 0.01 % by weight of the total nanoparticle dose were sequestered in the intestinal wall.

Another 5-day study was conducted in Wistar rats (3 animals per group) exposed to differentially sized TiO_2_ particles supplied by the Joint Research Center (JRC) Nanomaterials Repository. These reference materials consisted of anatase or rutile with mean particle diameter ranging from 6 to 90 nm. The particles were administered by gavage of aqueous suspensions at a daily dose of ~10 mg/kg for 5 consecutive days (Geraets et al., 2014[27]). The animals were sacrificed 24 h after the last dose and ICP-MS analyses of liver, spleen and mesenteric lymph nodes revealed marginal Ti levels, only occasionally exceeding the limit of detection. The fraction of nanoparticles absorbed and distributed to tissues was estimated by the study authors to be maximally 0.02 % by weight.

A 10-day repeated dose study was carried out by administration via gavage of an aqueous suspension containing rutile particles (mean size of 475 nm) to six Sprague-Dawley rats at a rate of 12.5 mg/kg per day (Jani et al., 1994[38]). SEM analyses and histologic inspection of tissues after the 10 days of dosing revealed the presence of aggregates or agglomerates of TiO_2_ particles in the gut-associated lymphoid tissue and mesenteric lymph nodes, with limited translocation to the liver, spleen, lung and the peritoneum. Quantitative Ti measurements by ICP-emission spectroscopy indicated that ~6.5 % of particles were absorbed, whereas the bulk of TiO_2_ remained in the feces. However, this calculation did not consider background Ti levels, thus overestimating absorption.

Another toxicokinetic study in Sprague-Dawley rats, carried out in part following the OECD test guideline 417, failed to detect any systemic uptake of food-grade anatase with a mean particle size > 100 nm (Farrell and Magnuson, 2017[25]). In this study, the particles were administered to the rats (6 animals per group) by incorporation into the rodent diet for 7 consecutive days at 200 mg/kg feed. The daily consumption of anatase particles was ~30 mg/kg body weight for males and ~20 mg/kg body weight for females. Thereafter, concentrations of Ti measured by ICP-atomic emission spectrometry in liver, kidney, muscle and blood were below the limit of detection or at the low level found in animals fed a control diet.

A 28-day study in Sprague-Dawley rats (6 animals per group) was conducted with two types of anatase nanoparticles administered by gavage at a dose of 250 mg/kg per day (Hendrickson et al., 2016[33]). The TiO_2_ particles were from the JRC Nanomaterials Repository (diameter of 5-10 nm) or from a commercial source (diameter of 20-25 nm). After the 28-day study period, animals were sacrificed and Ti was detected in tissues by atomic absorption spectroscopy. Following administration of the smaller particles, Ti was found in the brain, lung, heart, liver, kidney, spleen, small intestine, testicles and blood. After administration of the larger particles, Ti was found in the liver, kidney, spleen and small intestine, whereas Ti levels remained below the detection limit in the tissues of control animals. From these Ti concentrations, the FAF Panel estimated an oral bioavailability of ~0.0002 % (FAF Panel, 2021[24]).

In a 90-day study, TiO_2_ particles consisting of 80 % anatase and 20 % rutile (mean diameter of 26 nm) were administered orally to Sprague-Dawley rats (11 animals per group) at daily doses of up to 1042 mg/kg (Cho et al., 2013[16]). Following the 90-day administration period and analysis by ICP-MS, high Ti concentrations were found in the feces but no increased Ti levels were detected in liver, spleen, kidney and brain tissues even in the animals receiving the highest dose, thus indicating a very low absorption. In the blood taken at necropsy, the background Ti concentration of ~ 0.4 µg/g was minimally increased in males of the 521 and 1042-mg/kg dose groups.

In conclusion, the available toxicokinetic studies in rats indicate some limited systemic uptake of orally ingested nano-sized TiO_2_ particles but no evidence for a gastrointestinal uptake of ingested TiO_2_ particles in the submicron size range (diameter > 100 nm).

### 4.2 Oral bioavailability and biodistribution in mice

A single-dose experiment was conducted with food-grade anatase in C57BL/6 mice (4 animals per group). The test material with a mean particle diameter of 118 nm was suspended in water and administered by gavage at a dose of 40 mg/kg body weight (Coméra et al., 2020[17]). The absorption of particles was monitored by laser-reflective microscopy of intestinal tissue slides and blood smears. Compared to vehicle controls, the particle absorption in treated mice peaked at 4 h after gavage in jejunal and ileal villi before returning to basal levels at 8 h. In the jejunal Peyer's patches, more particles were detected at 8 h than at 4 h following administration. The mean diameter of particles in these tissues was ~700 nm. The amount of particles in the blood increased during the first 8 h after administration, at which time point the authors counted ~200 million particles per ml of blood against a background in control mice of ~50 million per ml. By extrapolation from the contents measured by IPC-MS, it was calculated that approximately 0.007 % of the administered Ti reached the intestinal mucosa within 4 h after administration.

Another oral route was used by dripping an aqueous suspension of anatase (mean particle diameter of 201 nm) into the mouth of NFR mice (22 animals per group), thus allowing for spontaneous swallowing of the test material with saliva (Talamini et al., 2019[72]). The dose was 5 mg/kg for 3 days per week over a period of 3 weeks, providing a mean exposure rate of 2 mg/kg/day. After this study period of 21 days, the animals were sacrificed and Ti concentrations in tissues were measured by ICP-MS. Only Ti levels in the large intestine and liver were higher in the treated mice compare to controls. Single-particle ICP-MS analyses demonstrated the presence of TiO_2_ particles in the wall of the large intestine. In its calculations, the FAF Panel estimated that 0.1 % of the total TiO_2_ dose was absorbed into intestinal wall and the liver (FAF Panel, 2021[24]).

A long-term biokinetic study was conducted by feeding C57BL/6 mice (6 animals per group) with diet containing anatase particles for 6, 12 and 18 weeks (Riedle et al., 2020[64]). The daily doses of anatase (mean diameter of 119 nm) were up to 100 mg/kg body weight per day. A control group was included without addition of anatase to the diet. After 18 weeks, the presence of particles in the cells of Peyer's patches was demonstrated by SEM coupled to energy-dispersive X-ray (SEM-EDX) detection, but no quantitative data were given.

Another long-term experiment was carried out in CD-1 mice (10 animals per group) by oral gavage of anatase particles in an aqueous suspension (mean diameters of 18 and 120 nm). The applied dose was 64 mg/kg per day (Gu et al., 2015[30]). Blood samples were taken at different times and analyzed by ICP-optical emission spectrometry, thus showing a peak Ti concentration of ~ 0.15 µg/ml (against a background of ~ 0.05 µg/ml) 1 h after administration of the 18-nm particles. However, no absorption into blood was measured with the 120-nm particles. The daily dosing with TiO_2_ particles was continued over 28 weeks. Thereafter, increased Ti levels were detected in the liver and pancreas, but again only in animals treated with the 18 nm-sized material.

In conclusion, the available toxicokinetic studies in mice lend support to a limited systemic uptake of orally ingested TiO_2_ particles. A small fraction of ingested submicron-sized TiO_2_ particles may hang around in the intestinal wall and in the gut-associated lymphoid tissue. Circumstantial evidence, such as the detection by laser-reflective microscopy and the measurement of Ti concentrations in tissue, suggests that spurious amounts of submicron-sized particles may also reach the blood and the liver.

### 4.3 Oral bioavailability in humans

Data obtained from human subjects lend, in part, support to a limited bioavailability of submicron-sized TiO_2_ after oral exposure. In one study, five volunteers ingested anatase particles as gelatin capsules (mean particle size of 160 nm) or as a powder (mean particle size of 380 nm). The anatase doses were 23 mg (corresponding to ~0.4 mg/kg body weight) and 46 mg (~0.75 mg/kg body weight), respectively (Böckmann et al., 2000[9]). Blood obtained before and at different times after administration, analyzed by ICP-atomic emission spectroscopy, revealed that background Ti levels ranged between 5.9 and 18.1 ng/ml. After TiO_2_ ingestion, Ti concentrations in the blood increased in all subjects. The highest Ti concentration of 109.9 ng/ml was detected in the blood of one volunteer 8 h after ingesting 46 mg of 160-nm anatase particles. Such a transient increase in blood Ti concentrations was not observed in a further study, where nine volunteers received a 5-mg/kg single oral dose of TiO_2_, consisting of anatase with a particle size of 15 nm or two distinct rutile particles in the submicron range (Jones et al., 2015[40]). The test materials were administered in gelatin capsules. Blood samples were collected before dosing and up to 48 h thereafter. ICP-MS revealed that, irrespective of the size, essentially none of the administered particles reached the blood.

Yet another human study involved seven volunteers, who ingested 100 mg of anatase (mean particle diameter of 260 nm) translating to ~1.5 mg/kg body weight (Pele et al., 2015[56]). The subsequent appearance of whole particles in the blood of five participants was suggested by dark field microscopy observations. This microscopic method could not be applied to the samples of two participants due to blood clotting. In parallel, Ti concentrations were measured in the same samples by ICP-MS. A peak Ti uptake into blood (~10 ng/ml against a background of ~ 1.5 ng/ml) was detected at 6 h after ingestion of the capsules. Thus, this report by Pele et al. (2015[56]) suggests that at least some of the absorbed Ti appears in the blood as whole particles.

The presence of particles was demonstrated in postmortem tissues of subjects deceased at the age of 56-104 years (Heringa et al., 2018[34]). In samples from liver and spleen, the authors detected TiO_2_ particles by SEM-EDX. The particle dimensions were from 85 nm (the smallest size detectable) to 720 nm. The highest Ti concentrations, measured by ICP-MS, were found in liver and spleen in the range of 40-80 ng/g tissue. According to the authors, almost all Ti was in the particulate form, as the concentration of particle-associated Ti overlapped with the total Ti concentration in the two organs. Similarly, particulate TiO_2_ was demonstrated in postmortem tissues by single-particle ICP-MS analysis of samples from subjects deceased at the age of 64-97 years (Peters et al., 2020[57]). Single particles as well as aggregates/agglomerates were visualized by SEM-EDX. The particulate Ti content ranged from 0.01 to 1.8 mg/kg with the highest concentrations found in the intestinal wall. The size of most of these particles was in the range of 65-400 nm. These two studies illustrate that TiO_2_ particles appear at low concentrations in internal organs. The authors propose that the TiO_2_ detected in human liver, kidney and spleen may originate from food-associated exposures, but alternative sources are *inter alia* pharmaceutical formulations, sunscreen cosmetics, dental prostheses or other Ti implants.

In conclusion, observations in humans provide further support for a limited systemic uptake of orally ingested TiO_2_ particles. Again, circumstantial evidence, such as dark field microscopy and the measurement of Ti concentrations in tissue, suggests that spurious amounts of dietary submicron-sized particles may reach the blood circulation.

## 5. Rodent Toxicity Studies

Only toxicity studies conducted with food-grade or pigment-grade TiO_2_ particles with characteristics comparable to E171 were considered for the purpose of this review. Routes of exposure other than oral intake were judged not to be relevant for the risk assessment of E171 as food additive. All toxicity studies considered in this review are included in Table 2(Tab. 2).

### 5.1 Subacute toxicity

None of the acute toxicity studies with food-grade or pigment-grade TiO_2_ particles, identified in the literature, fulfill minimal particle characterization requirements including the determination of shape, composition, crystalline form and primary size. In any case, repeated dosing is considered more relevant than single dose experiments for the assessment of a continued dietary exposure. A 21-day subacute toxicity study, already described in the bioavailability section 4.2, was conducted by oral administration of anatase (mean particle diameter of 201 nm) to NFR mice (22 animals per treatment group; Talamini et al., 2019[72]). The rate of anatase administration was 2 mg/kg/day and it had been estimated that 0.1 % of the total TiO_2_ dose is systemically absorbed (FAF Panel, 2021[24]). This exposure did not affect body weight, feed intake or the weight of organs. There were, in some tissues, treatment-related changes in superoxide and interleukin-1β levels that may be interpreted as adaptive responses rather than toxicologic adversities. Also, the authors of the study detected inflammatory areas in the liver of exposed mice, but these reactions were not accompanied by histologic and biochemical endpoints that would provide evidence for a toxicologically relevant tissue injury.

A 28-day subacute toxicity study was carried out in line with the OECD test guideline 407 using rutile particles with a mean diameter of 173 nm. This test material was suspended in water and administered by oral gavage to Sprague Dawley rats (5 animals per group) at the daily dose of 24000 mg/kg body weight (Warheit et al., 2015[81]). There were no test item-related effects on mortality, feed intake, body weight, clinical signs, hematology, clinical chemistry, urine analysis, organ weights, gross pathology or histopathology. Granular aggregates, indicative of TiO_2_ clusters, were observed in hematoxylin/eosin-stained sections of the intestinal mucosa and associated lymphoid tissue, but without cellular reactions. These microscopic findings related to the presence of test particles in gut and lymphoid tissues were not considered adverse.

In conclusion, subacute studies indicate that oral exposure to E171 do not elicit toxicity in rodents, even when tested at high doses.

### 5.2 Subchronic toxicity

A 90-day study compliant with the OECD test guideline 408 was carried out with food-grade anatase particles displaying a mean diameter of 150 nm (Han et al., 2021[31]). The particles were suspended in water and administered via gavage to Sprague Dawley rats (groups of 20 animals) at doses of up to 1000 mg/kg body weight per day. There were no test item-related effects on mortality, body weight, clinical signs, hematology, serum clinical chemistry, urine analysis, hematology, gross pathology and organ weights. The authors reported a slightly increased feed intake and a marginal reduction of lymphocyte counts in the males of the highest dose group. Also, the histopathologic analysis of animals in the high-dose group revealed that particles accumulated in cells lining the stomach wall. TEM images showed that E171, again in the highest dose, induced lamellar body-like structures in cells of the colon epithelium. Such structures have been associated with the defense against foreign substances and, hence, may be regarded as an adaptive response to high particle doses. In fact, the FAF Panel at EFSA concluded that the administration of E171 in doses of up to 1000 mg/kg per day did not induce adverse effects in this 90-day study (FAF Panel, 2021[24]).

A 196-day study was conducted in mice at a test item dose of 64 mg/kg body weight per day. CD-1 mice (10 animals per group) were exposed to anatase particles with mean diameter of 120 nm by oral gavage of an aqueous suspension (Gu et al., 2015[30]). In this study, which has already been described in the bioavailability section 4.2, no systemic uptake of the 120-nm particles could be detected. Accordingly, the thorough inspection of animals after termination of the study did not reveal any gross pathologic or histologic changes.

In conclusion, subchronic toxicity studies indicate that oral exposure to E171 does not elicit adverse effects in rodents, even when tested at the limit dose of 1000 mg/kg/day.

### 5.3 Chronic toxicity

Long-term effects of TiO_2_ have been studied mainly upon airway exposure. The International Agency for Research on Cancer (IARC) concluded that there is inadequate evidence from epidemiologic studies to assess whether TiO_2_ dust causes cancer in humans. Conversely, there is sufficient evidence for carcinogenicity in experimental animals in view of respiratory tract tumors induced by prolonged inhalation in rats (Lee et al., 1985[42]; Baan et al., 2006[4]). As a consequence, TiO_2_ particles are classified as a Group 2B carcinogen (IARC, 2010[37]).

Oral 2-year studies were carried out in the frame of the National Toxicology Program (1979[51]). The test material consisted of pigment-grade anatase designated Unitane^®^ 0-220. The particle size is not specified, but from the optical characteristics (white color) a mean particle or cluster diameter in the 200-300 nm range can be assumed. This test material was incorporated into the feed of Fisher 344 rats (groups of 50 males and 50 females) at daily doses of 1125 and 2250 mg/body weight (for males) and 1450 and 2900 mg/kg (for females). With the exception of the appearance of white feces, no clinical effects related to TiO_2_ exposure were observed. The test substance did not influence survival. At the end of the 103-week exposure period, the organs of surviving animals were analyzed macro- and microscopically. There was an increased frequency of hyperplastic bile ducts in the males, which were not considered to be treatment-related. With regard to tumors, it was noticed that, in females, the combined incidence of adenomas and carcinomas of the thyroid was increased from 2 % in the control group to 14 % in the high-dose group. This difference was not statistically significant upon a Bonferroni correction for multiple comparisons. It was, therefore, concluded that the oral TiO_2_ administration is not carcinogenic. The relevance of thyroid tumors in rats for human cancer risks is in any case questionable due to the higher susceptibility of the rodent thyroid to develop neoplastic sequelae (Alison et al., 1994[1]).

The same Unitane^®^ 0-220 material was incorporated into the diet of B6C3F1 mice (groups of 50 males and 50 females) at daily doses of 3250 and 6500 mg/kg body weight (for males) and 4175 and 8350 mg/kg (for females). With the exception of white feces, there were no clinical signs that could be linked to TiO_2_ administrations. The test item did not impinge on the survival of male mice but, in the females of the high-dose group, only 66 % survival was reported at the end of the 104-week study period compared to 90 % survival in controls. No accompanying findings were reported that would explain this gender-specific difference. All surviving animals were sacrificed at the end of the 103-week period for macroscopic and microscopic examinations. There was an increased incidence of hepatocellular carcinomas in the males of the high-dose group. However, the test laboratory noted that this observed occurrence of liver cancer remained within the range of historical reference controls and, therefore, it was concluded that the oral TiO_2_ administration is not carcinogenic in mice (National Toxicology Program, 1979[51]).

In conclusion, chronic toxicity studies indicate that oral exposure to E171 does not elicit carcinogenicity or other long-term adverse effects in rodents, even when tested at high doses.

### 5.4 Reproductive and developmental toxicity

Only few reproductive studies are available for food-grade or comparable pigment-grade TiO_2_ particles. A prenatal developmental study was carried out in pregnant rats, following the OECD test guideline 414, to evaluate three pigment-grade anatase and rutile particles (Warheit et al., 2015[80]). The mean primary size of these particles was in the range of 153-213 nm. Pregnant Sprague-Dawley rats were treated by gavage on gestation days 6-20, pregnant Wistar rats were treated by gavage on gestation days 5-19. The oral dose rate in both strains was up to 1000 mg/kg body weight per day. At the end of each exposure period, just before the expected parturition, the rats were sacrificed for caesarean sections and careful inspection of the dam and fetuses. This study revealed no evidence of maternal or developmental toxicity at any dose level. In another reproduction toxicity study, conducted according to OECD test guideline 421 and summarized in Warheit et al. (2015[80]), Sprague-Dawley rats were dosed with daily gavages of pigment-grade TiO_2_ at 1000 mg/kg body weight. The protocol involved exposure of the males for 40 days (beginning from 2 weeks before the mating period) and exposure of the females for 2 weeks before mating, during gestation and until day 4 after delivery. This study did not disclose any reproductive or developmental toxicity.

An extended one-generation reproductive toxicity (EOGRT) study submitted to EFSA was carried out according to OECD test guideline 443, using food-grade anatase with a mean particle diameter of ~100 nm (FAF Panel, 2021[24]). The particles were administered to Sprague-Dawley rats (40 animals per group) as part of their diet at daily doses of up to 1000 mg/kg body weight. In the F0 generation, exposure with anatase took place from 10 weeks before mating until weaning of the F1 generation. The F1 generation was treated from weaning until postnatal days 4-8 of their offspring. The animals in the higher dose groups showed pale feces but no treatment-related adverse effects were observed at any generation. The endpoints examined included clinical signs, body weight, food and water consumption, hematology, clinical biochemistry, urine analysis, diverse hormones, estrus cycle, sperm parameters, male and female fertility and macroscopy/microscopy of organs. No effects were observed on pre- and postnatal development.

In conclusion, the evidence is increasing that dietary E171 does not cause reproductive toxicity in laboratory animals.

## 6. Studies on Genotoxity

The question of whether TiO_2_ generates DNA damage has been tested using a range of different assays. The canonical reverse mutation test in bacteria (Ames test) is not suitable because the rigid prokaryotic cell wall would prevent the uptake of particulate test materials. Conflicting findings (both positive and negative outcomes) were reported from *in vitro* tests carried out in rodent or human cells, aiming at the detection of DNA strand breaks, gene mutations, chromosomal deletions or other chromosomal aberrations, as well as micronuclei or sister chromatid exchanges (reviewed by FAF Panel, 2021[24]). The interpretation of *in vitro* assays is uncertain because the effects, if any, may be secondary to cytotoxic or apoptotic responses of cultured mammalian cells exposed to high particle concentrations.

Instead, *in vivo* genotoxic responses are likely a consequence of the ability of TiO_2_ to generate ROS, thus giving rise to oxidative stress, tissue inflammation and oxidative DNA damage (Trouiller et al., 2009[75]; Shukla et al., 2014[69]; Shi et al., 2015[68]). Inflammatory reactions to TiO_2_ are characterized by macrophage and neutrophile infiltration as well as the release of chemokines and cytokines (Dankovic et al., 2007[19]; Olmedo et al., 2008[54]; Chen et al., 2009[14]). There are at least three major determinants of oxidative tissue damage. First, the oxidative stress in tissues depends on the oral bioavailability, i.e, the extent of internal exposure to TiO_2_ particles after ingestion with food. Second, the severity of oxidative stress is a function of the mean particle size defining the reactive particle surface relative to particle mass. These two parameters are fundamentally different between TiO_2_ nanoparticles and food-grade counterparts, because nanoparticles display both a higher bioavailability and greater outer particle surface. Therefore, data obtained with TiO_2_ nanoparticles are of questionable relevance for the genotoxicity assessment of E171. Third, as already pointed out, it can be expected that the nanoparticle fraction of E171 aggregates or agglomerates with concomitantly present larger particles, such that the resulting clusters lose nanomaterial properties. This view is supported by the finding that anatase and rutile particles with mean diameter of 75 nm or higher do not display *in vivo* genotoxic properties - at least as measured by the micronucleus test according to OECD guideline 474 - after acute or subacute oral administration in laboratory animals (Chen et al., 2014[15]; Donner et al., 2016[23]). The notable exception is a report suggesting that orally administered TiO_2_ particles of 117 nm in diameter lead to DNA strand breaks - determined by single-cell gel electrophoresis (comet) assays - in peripheral blood cells of mice (Murugadoss et al., 2020[50]). However, the relevance of this study is limited by the lack of a dose dependence between the lowest dose and a 25fold increased exposure in the high-dose group, and by the fact that no internationally recognized protocol was used for the comet assay.

The above considerations were not shared by the FAF Panel at EFSA in their recent evaluation of the genotoxic potential of E171 (FAF Panel, 2021[24]). With regard to *in vivo* genotoxicity studies, the EFSA experts also evaluated the aforementioned oral study carried out by Chen et al. (2014[15]), whereby Sprague-Dawley rats (7 animals per group) were exposed to anatase with a mean particle size of 75 nm. Gavage administrations were carried out for 30 days at daily doses of up to 200 mg/kg body weight per day. Thereafter, a micronucleus test carried out on bone marrow erythrocytes revealed no increased DNA damage in TiO_2_-treated animals compared to untreated controls. The FAF Panel considered five other reports involving micronucleus tests, chromosomal aberration and comet assays in rodents following the oral route of exposure. In a subacute study, an aqueous suspension of anatase nanoparticles with a primary size of 20-50 nm was administered by gavage to Swiss albino mice (5 animals per group) for 14 days (Shukla et al., 2014[69]). The daily TiO_2_ doses were up to 100 mg/kg body weight. Following the exposure period, the authors observed an accumulation of mononuclear cells in the liver of the animals that were exposed to the highest dose. A comet assay was conducted on single-cell suspensions of the liver and a micronucleus test was carried out on bone marrow erythrocytes. The comet assay revealed increased DNA damage frequencies in all treatment groups compare to controls, whereas the micronucleus test was positive only in the mice treated with the highest anatase dose of 100 mg/kg/day. In another subacute study, an aqueous suspension of rutile nanoparticles with a mean primary size of 25 nm was administered by gavage to Swiss albino mice (5 animals per group) for a period of 28 days (Manivannan et al., 2020[45]). The daily doses were up to 0.8 mg/kg body weight. After termination of the study, single cells isolated from different tissues were analyzed by comet assay and the bone marrow was scrutinized for chromosomal aberrations. Following the nanoparticle treatments, the authors observed an increased frequency of DNA breaks in cells isolated from liver, spleen, thymus, lymph node and bone marrow. There was also an accumulation of chromosomal aberrations (mainly chromatid breaks) in metaphase cells of the bone marrow. In a 60-day study, an aqueous suspension of anatase nanoparticles with a primary size range of 5-12 nm was administered by gavage to Wistar rats (6 animals per group). The daily doses were up to 200 mg/kg body weight (Grissa et al., 2015[29]). After the exposure period, peripheral leukocytes were subjected to the comet assay and bone marrow cells to the micronucleus test. There was an increased frequency of strand breaks and micronuclei in the TiO_2_-treated animals. Two additional genotoxicity studies with nanoparticles evaluated by the FAF Panel (Sycheva et al., 2011[71]; Chakrabarti et al., 2019[13]) are not included in the present review because of insufficient particle characterization (Table 1(Tab. 1)). Studies based on the administration of TiO_2_ by intraperitoneal or intravenous injections are also not considered here because parenteral routes lack relevance for the risk assessment of food-associated particles.

To sum up, the appraisal of genotoxicity studies - based on scientific quality, plausibility and relevance - leads to the conclusion that TiO_2_ particles with mean diameter of 75 nm or higher do not exert *in vivo* genotoxicity following oral ingestion.

## 7. Local Effects in The Gastrointestinal Tract

One frequently asked question is whether E171 may influence the bacterial community in the gut lumen. Various tests carried out *in vitro* and *in vivo* showed that food-grade TiO_2_ is able to cause shifts in intestinal bacteria populations (Pinget et al., 2019[59]; Yan et al., 2020[83]). For example, the intestinal flora was examined in mice exposed to anatase particles for 56 days (Cao et al., 2020[11]). In these experiments, the feed of C57BL/6 mice (15 animals per group) contained TiO_2_ nanoparticles (mean diameter of 33 nm) or food-grade TiO_2_ (mean diameter of 112 nm) at a concentration of 1000 mg/kg feed. There were no overt toxic effects but the authors observed changes in the bacterial composition of feces at the end of the study period, i.e., an increased abundance of bacteria of the phylum Firmicutes accompanied by a reduction of Bacteroidetes, including representatives of the genera *Bifidobacterium* and *Lactobacillus*. The nanoparticles elicited stronger effects on microbiota composition than E171, and obese mice were more susceptible to these effects than the non-obese. The interpretation of such findings is problematic as it is not yet known what changes in the microbiota are to be considered adverse.

Another concern is that E171 may impose oxidative stress on the epithelial cells lining the gastrointestinal tract, leading to cell death, chronic inflammation and a predisposition to gastrointestinal cancer. The consequences of food-grade anatase (primary particle size of 118 nm) on the intestinal mucosa were monitored in Wistar rats (Bettini et al., 2017[6]). In one experiment, the test material suspended in water was administered by daily gavage at 10 mg/kg body weight for 7 days in the absence of any additional treatment (10 animals per group). The authors observed an internalization of TiO_2_ particles not only in the lymphoid tissue of Peyer's patches but, to a minor extent, also in the mucosa of the colon and in the liver. In a second experiment, the rats (12 animals per group) were pretreated with the tumor initiator 1,2-dimethylhydrazine (DMH) followed by exposure to the food-grade anatase - through the drinking water - for 100 days. In this tumor model, anatase particles at 10 mg/kg/day increased the frequency of a pre-carcinogenic stage known as aberrant crypt foci (appearing as abnormal glands), from around ~475 per colon in the rats treated with DMH alone to ~550 per colon in rats receiving both DMH and the particles. Another experiment revealed that 4 out of 11 animals developed aberrant crypt foci upon anatase particle exposure at 10 mg/kg/day even in the absence of a DMH pretreatment. No aberrant crypt foci were detected in the colon of 12 control animals. This exposure to anatase particles also led to a slightly increased expression of the inflammatory markers TNF-α, IL-1β, IL-8 and IL-10 in the colon. The authors concluded that food-grade anatase induces local inflammation in the mucosa that promotes preneoplastic colonic lesions. Retrospectively, this conclusion may appear premature in view of the rather low number of animals and the lack of historic control data as a reference. This effect of anatase particles in generating aberrant crypt foci was not replicated in later investigations (Blevins et al., 2019[8]; FAF Panel, 2021[24]).

In the report by Blevins et al. (2019[8]), food-grade anatase (primary particle size of ~115 nm) was administered to Wistar Han rats as part of their diet at daily doses of up to 374 mg/kg for 7 days (5 animals per group) and at daily doses of up to 267 mg/kg for 100 days (15 animals per group). For the 100-day study, animals were pretreated with a single injection of DMH. No mortality or signs of toxicity occurred and, at the end of the study periods, there were no changes in food intake, body weight or organ weights. Additionally, no local inflammatory responses in the intestinal mucosa and no changes in the frequency of preneoplastic lesions in the form of aberrant crypt foci were detected. The FAF Panel noted some technical limitations as to the histopathologic examination of tissues that may have influenced the count of aberrant crypt foci. However, this same endpoint was included in the EOGRT study submitted to EFSA (see section 5.4). In a satellite group of that study (20 animals per group), submicron-sized anatase particles at doses of up to 1000 mg/kg per day did not induce aberrant crypt foci in the colon (FAF Panel, 2021[24]).

## 8. Conclusion

Taken together, the available reports indicate a size-dependent biokinetic behavior whereby TiO_2_ particles in the nano-sized range are absorbed more effectively from the gastrointestinal tract than larger submicron-sized counterparts (section 4). There is only a marginal systemic absorption of orally administered TiO_2_ displaying mean primary sizes > 100 nm. In many studies, a systemic distribution was inferred from Ti measurements without demonstrating the particulate form of residues in blood or tissues. After oral administration in laboratory animals, the presence of TiO_2_ particles has been demonstrated in the intestinal mucosa, in submucosal layers of the intestinal wall and, to a minor extent, in the liver. Only trace amounts of TiO_2_ particles reach the blood and internal organs.

A commonly ignored aspect is that TiO_2_ particles readily aggregate or agglomerate, thus increasing the overall particle size (section 2 and Figure 1(Fig. 1)). As a consequence, the nano-sized fraction of E171 merges into submicron- or even micron-sized clumps thereby nullifying the “nano” connotation of this food additive. Indeed, TiO_2_ particles found in the gastrointestinal lumen, in the intestinal mucosa, blood and tissues are predominantly in an aggregated or agglomerated form and it is questionable whether small traces of large TiO_2_ clusters display adverse effects. This view is confirmed by a broad range of toxicity studies indicating that E171 and comparable pigment-grade particles fail to elicit adverse effects upon oral ingestion in laboratory animals, even when tested at high doses (section 5 and Table 2(Tab. 2)). In one report, E171 had been associated with preneoplastic effects in the intestinal mucosa, but these findings were not reproduced in follow-up studies (section 7). For the same reason, it is also inappropriate to assume that genotoxicity findings obtained by exposing laboratory animals to TiO_2_ nanoparticles translate to an analogous hazard associated with E171 formulations. In fact, TiO_2_ particles with a mean diameter of 75 nm or higher generated negative results in the *in vivo* micronucleus test according to OECD standards (section 6).

The case of E171 illustrates how the risk assessment of manufactured particulate food additives is potentially biased towards purported hazards of particles in the nano-sized range. As a consequence of this perceived “nano” connotation, there is a danger that the risk assessment is influenced and biased by insufficient data quality (for example due to poor characterization of the test materials, see Table 1(Tab. 1)), by erroneous assumptions (for example by postulating that the biological effects of a nano-sized tail can be assessed regardless of buffering effects of concomitantly present submicron-sized particles, see Figure 1(Fig. 1)), by the use of study protocols that do not follow internationally recognized guidelines (for example on how to conduct and interpret comet assays, see section 6) or by isolated findings that due to their preliminary nature with a small number of animals fail reproduction by independent laboratories (such as the detection of intestinal preneoplastic lesions, see section 7).

Even though the health risk of E171 may have been overestimated as a consequence of the above-described bias towards a fictional “nano” hazard, it may still be proportionate to abandon its use as a food additive in view of remaining uncertainties as to possible long-term effects on the intestinal mucosa (Bischoff et al. 2021[7]), perhaps exacerbating chronic inflammatory bowel disease, while there is no demonstrable benefit for the consumers. However, the paradigm of E171 highlights that it will be important that such a biased assessment is not applied to newly engineered products that may confer true benefits to the consumer by improving nutritional benefits, quality, freshness, traceability or sustainability of food on the market. A safety assessment biased towards a factionary “nano” hazard would severely dampen innovation in the European food industry.

## Declaration

### Conflict of interest

The authors declare that they have no conflict of interest.

## Figures and Tables

**Table 1:**
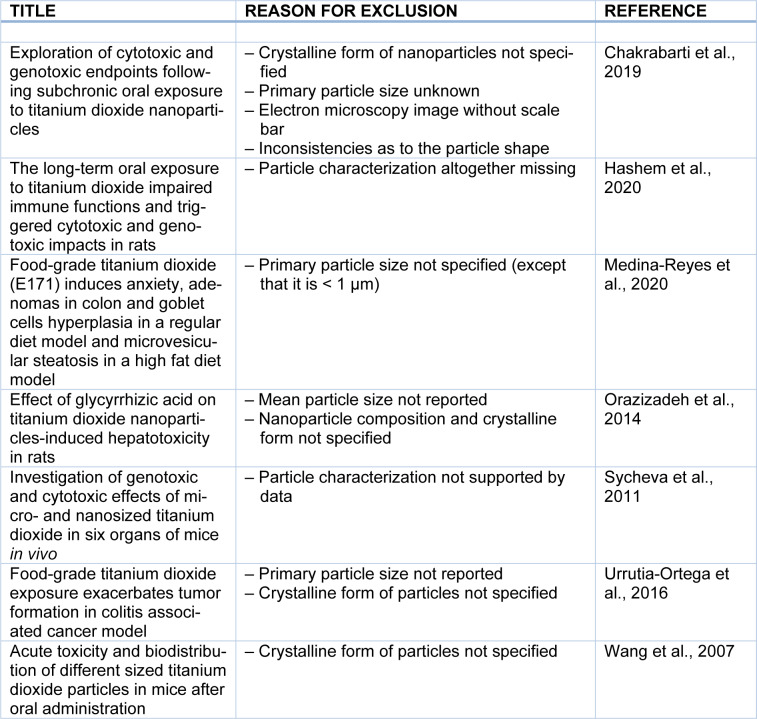
Studies related to the safety of TiO_2_ materials not considered in this review due to incomplete particle characterization

**Table 2:**
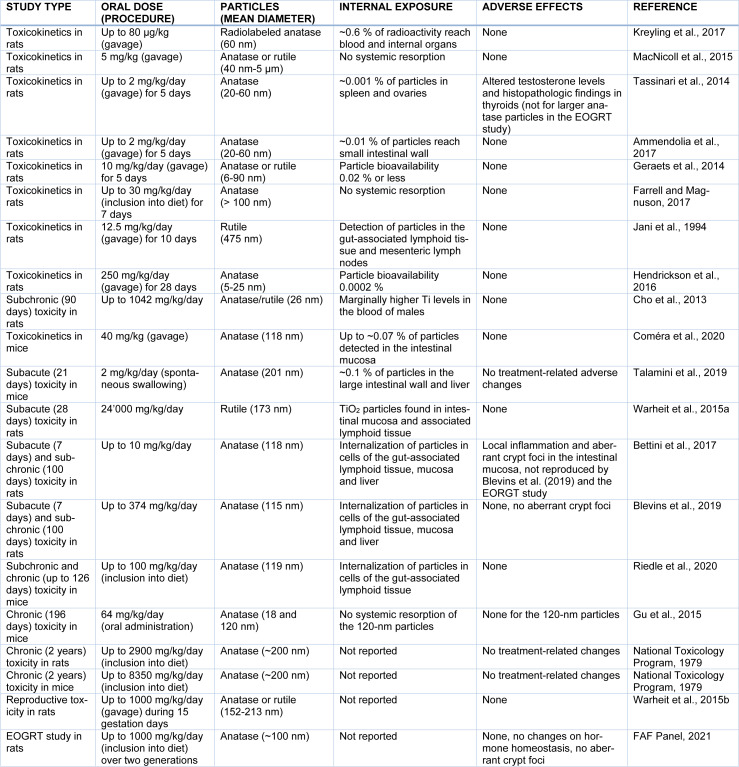
Overview of oral toxicokinetic and toxicodynamic studies in rodents relevant for the risk assessment of E171
